# Differential Diagnosis of Inflammatory Arthritis from Musculoskeletal Ultrasound View

**DOI:** 10.2478/rir-2022-0010

**Published:** 2022-07-06

**Authors:** Yasushi Kondo, Yuko Kaneko, Tsutomu Takeuchi

**Affiliations:** 1 Department of Internal Medicine, Division of Rheumatology, Keio University School of Medicine, Shinjuku, Tokyo 160-8582, Japan

**Keywords:** musculoskeletal ultrasonography, inflammatory arthritis, differential diagnosis, synovitis

## Abstract

Diagnostic imaging in rheumatology has evolved over the centuries, and novel imaging modalities, including musculoskeletal ultrasonography (MSUS) and magnetic resonance imaging (MRI), are being widely used in the 21st century. With the increase in availability of molecular target–specific therapies, including biologic agents and Janus kinase (JAK) inhibitors, the therapeutic outcome of inflammatory arthritis has changed, and early and accurate diagnosis of inflammatory rheumatic diseases has become more important. Given this situation, MSUS, which is a portable, convenient, noninvasive, and cost-effective imaging technique, plays an important role in the diagnosis of rheumatic diseases. MSUS can be used to detect subclinical inflammation and to accurately determine the distribution of joint involvement and inflammation sites in each joint. Definitive diagnosis for patients with early arthritis should be made after noting their history and performing clinical examination, laboratory testing, and additional procedures. However, MSUS is an extension of physical examination and it can provide a further opportunity and motivation to consider differential diagnoses rather than a conclusive diagnosis. This review aims to describe the usefulness of MSUS in differential diagnoses of the phenotype of early inflammatory arthritis.

## Introduction

Diagnostic imaging in rheumatology has evolved over the centuries. Classical radiography was first used in 1890. it was recognized as the gold standard for the evaluation and diagnosis of structural damage associated with rheumatoid arthritis (RA) in 1987. Musculoskeletal ultrasonography (MSUS) and magnetic resonance imaging (MRI) were first used in the 1970s. Since then, they have been continuously developed until 2000 and are widely used in the 21st century.^[1]^ The extensive use of these imaging modalities in rheumatology is mainly attributed to the development of therapeutic strategies for RA. In the 21st century, the advent of many biologic agents and JAK inhibitors has resulted in therapeutic innovations for RA. As a result, the main target of RA treatment has changed to clinical, structural, and functional remission. In addition, early RA diagnosis is critical for achieving remission.^[2,3,4,5]^ Further, these new therapies, including biologic agents and JAK inhibitors, are available not only for RA but also for psoriatic arthritis (PsA), spondyloarthritis (SpA) associated with inflammatory bowel disease, and crystal-induced arthritis; it has also been clarified that the target molecules are different in each inflammatory arthropathy.^[6,7,8,9,10,11]^ Therefore, the differentiation of inflammatory rheumatic diseases is becoming increasingly important for physicians. In this situation, distinguishing early inflammatory rheumatic disease from various inflammatory rheumatic diseases using only routine classical evaluations, including physical examination, laboratory testing, and radiography, is sometimes challenging.^[12]^ For instance, radiographs can be used to visualize structural damage, including bone erosion, joint space narrowing, mutilation, subluxation, and ankylosis; however, they cannot detect direct joint inflammation, such as synovitis and tiny bone erosions. In contrast, ultrasound and MRI can provide direct visualization of subclinical active inflammation or tiny bone erosions that cannot be identified via physical examination and classical radiographs in the early phase of inflammatory arthritis.^[13,14,15]^ Therefore, this paper aims to review the usefulness of ultrasound in formulating differential diagnoses of the phenotype of early inflammatory arthritis.

### Characteristics of MSUS

**Figure 1:**
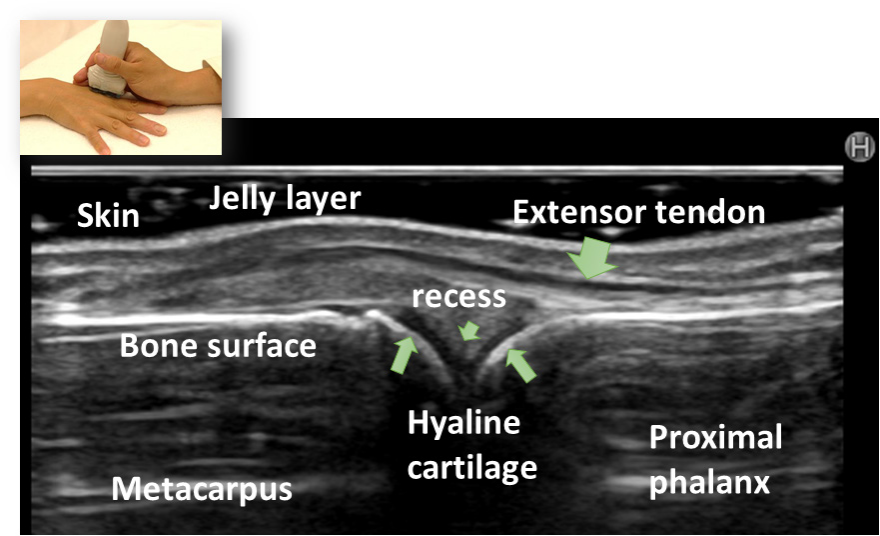
Ultrasonography can visualize comprehensive musculoskeletal tissue (healthy, dorsal metacarpal phalangeal joint). Ultrasound generates high-resolution images depending on the reflection rate of each musculoskeletal anatomical structure and almost completely reflects the bone surface. The joint cavity could not be identified in healthy subjects.

Joint ultrasonography is a portable, convenient, noninvasive, and cost-effective imaging technique that can be used to visualize musculoskeletal tissues, including the skin, nails, tendons, ligaments, entheses, synovia, bursae, cartilage, and bone surface (Figure 1). It can provide high-resolution images of anatomical changes and damage to joint and soft tissues induced by inflammatory or degenerative conditions in various rheumatic diseases, such as RA, osteoarthritis, SpA, crystal-induced arthritis, and septic arthritis, and connective tissue diseases, such as systemic lupus erythematosus (SLE), systemic sclerosis, inflammatory myositis, Sjögren's syndrome, and systemic vasculitis.^[12,13,14,15]^

The different characteristics of the novel imaging modalities for rheumatic diseases, including MSUS and MRI, are summarized in Table 1. The advantages of MSUS over other imaging techniques include visualization of the joint cavity and soft tissue involvement, cost-effectiveness, availability, absence of radiation, multiplanar imaging capability, and provision of real-time dynamic assessment of joints. Additionally, MSUS is a useful tool for guiding invasive procedures. It enables accurate and precise needle positioning and is a safe approach for patients requiring synovial fluid aspiration, injection therapy, and/or biopsy.^[12,13,14, 16]^ MRI is a more sensitive imaging modality for visualizing inflammation and damage, and in particular it is uniquely useful in detecting bone inflammation; however, it is expensive, not easily available, time-consuming, and restricted to a limited anatomical area per examination.

**Table 1:** Comparison of the characteristics of the imaging modalities MSUS and MRI in rheumatology

**Characteristics**	**MSUS**	**MRI**
**Accessibility** (convenience, handy, time, etc.)	Good (but, time-consuming)	Poor
**Scanning area**	Multiple	Single site (depends on the coil)
**Examination cost**	Low cost	Expensive
**Detectable pathological findings**		
Synovitis	º	º
Tenosynovitis	º	º
Bone edema (Osteitis)	×	º
Bone erosion	º	º
Cartilage	º	º

MSUS, musculoskeletal ultrasonography; MRI, magnetic resonance imaging; º, detectable; ×, not detectable.

### Definition of Ultrasonographic Evaluation in “Active Synovitis”

Synovitis is the main feature of inflammatory arthritis and is characterized by intra-articular synovial hypertrophy, which is detected using gray-scale ultrasonography (GSUS). Its ultrasonographic features include abnormal thickness, hypoechogenicity relative to subdermal fat, and poor compressibility.^[13]^ We also evaluated synovitis activity using power or color Doppler ultrasound (PDUS) to assess vascularity. PDUS has been used to determine the activity and specificity of joint inflammation.^[17]^ Both synovial hypertrophy and vascularity detected by MSUS were significantly associated with local inflammatory cytokine and chemokine levels, including Interleukin (IL)-6, IL-1β, IL-10, IL-17, and granzyme B.^[18]^ Vascular endothelial growth factor (VEGF) and vascular chemokine fractalkine were specifically correlated with synovial vascularity (PDUS). Thus, active synovitis refers to synovial hypertrophy detected on GSUS and vascularity detected on PDUS in untreated patients with RA. In contrast, the association between synovial hypertrophy and cytokines or growth factors was diminished in treated patients with RA, although PDUS and hypoechogenicity of the synovium remained correlated with IL-6 and VEGF, which were associated with synovial inflammatory cell infiltration and vascularity of the corresponding synovial tissue obtained by US-guided biopsy. Therefore, quality assessment of the synovium, including PDUS and hypoechogenicity, rather than synovial hypertrophy itself, seemed to be more important for evaluating the activity of joint inflammation in treated patients with RA.

### Use of MSUS in Differentiating Inflammatory Arthritis

The European League Against Rheumatism (EULAR) recommends that a definitive diagnosis for patients with early arthritis should be made after noting their history and performing clinical examination, laboratory testing, and additional procedures. Clinical examination remains the cornerstone for detecting synovitis, and subsequently, it may be confirmed by MSUS.^[19]^ In our opinion, novel imaging techniques, including MSUS and MRI in rheumatology, are an extension of clinical examination and provide a further opportunity and motivation to consider differential diagnoses via accurate information of active inflammation or structural abnormalities rather than a conclusive diagnosis in patients with inflammatory arthritis. MSUS can be used to accurately reveal the distribution of joint involvement by detecting subclinical joint inflammation and to identify the main affected part of the joint (tendon, enthesis, ligament, and bursae). This imaging technique not only reveals inflammation-related anomalies but also depicts non-inflammatory changes, such as bone and cartilage alterations and crystal deposition.

### Distribution reclassification

In the early phase of inflammatory rheumatic disease, it may be difficult to detect joint inflammation using ordinal physical examination alone. The value of ultrasound in identifying subclinical synovitis has been demonstrated by the detection of synovitis in asymptomatic joints of patients with early oligoarthritis, which has led to the reclassification of polyarthritis. Ultrasound and MRI have been used to detect joint inflammation more frequently than clinical examinations. The mean detection rates for synovitis in the hand and wrist were 2.18-fold and 2.20-fold for ultrasound and MRI, respectively.^[13, 20]^ The inter-observer reliability for detecting the presence of active inflammation between examiners was more accurate in MSUS than in physical examination.^[21]^ Additionally, based on our data, if positively enhanced MRI is the gold standard for active synovitis, MSUS power Doppler findings are concordant with MRI findings. However, this was not the case for physical examination (Table 2). Furthermore, ultrasound assessment improves the accuracy of the 2010 ACR/EULAR RA criteria in detecting subclinical active synovitis, especially in patients with suspected RA with a score of 3–7 according to the 2010 ACR/ELAR RA criteria.^[22]^

**Table 2:** Discrepancy of “active synovitis” between MRI, physical examination, and MSUS

**Assessed joint**	**Gold standard**	**Comparison**	**κ value**
PIPJ	MRI vs	physical examination	0.20
		MSUS (PD ≥ 1)	0.52
MCPJ	MRI vs	physical examination	0.22
		MSUS (PD ≥ 1)	0.84
**Wrist**	MRI vs	physical examination	0.31
		MSUS (PD ≥ 1)	0.70

MSUS, musculoskeletal ultrasonography; MRI, magnetic resonance imaging; PIPJ, proximal interphalangeal joints; MCP, metacarpophalangeal joints; PD, Power Doppler.

### Clarification of the main affected sites of the joint

**Figure 2:**
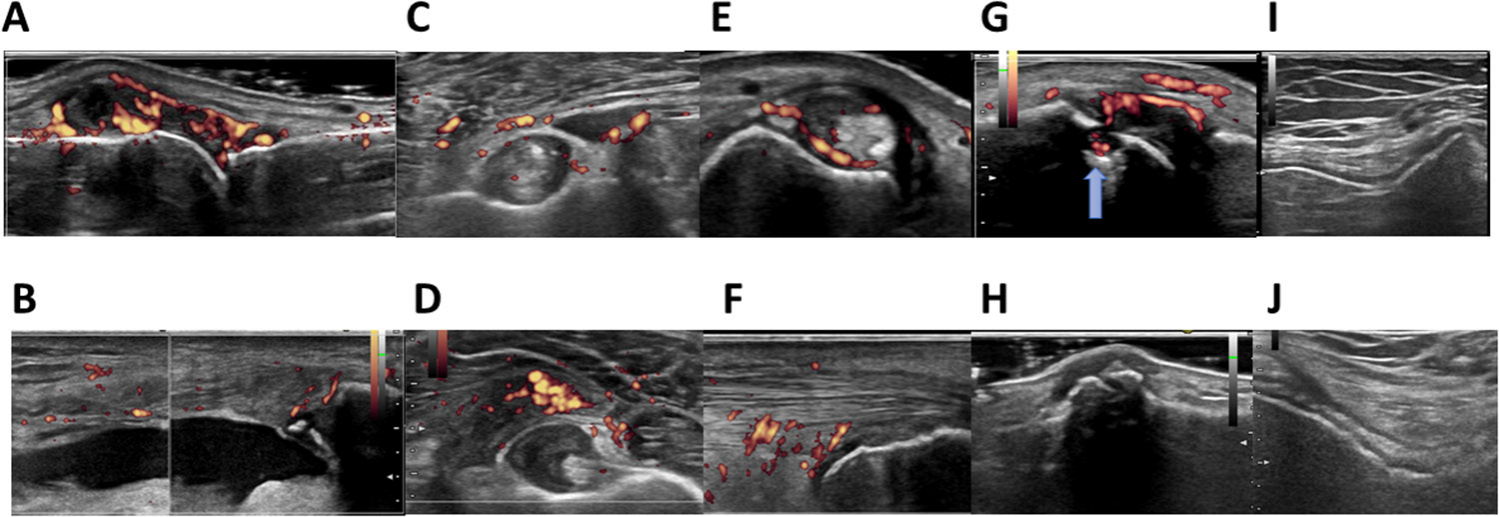
Typical pathological findings of MSUS in inflammatory arthritis. (A) Active synovitis with synovial hypertrophy and power Doppler signals. (B) Synovial fluid retention in the knee joint. (C, D) Bursitis in a patient with PMR and RA. (E) Tenosynovitis in the extensor carpi ulnaris tendon. (F) Achilles tendon enthesitis. (G) Bone erosion in a patient with active RA. (H) Osteophyte on the proximal inter-phalangeal joint in an osteoarthritis patient. (I) Cartilage loss. (J) Crystal deposits on the hyaline cartilage of the knee joint in a patient with calcium pyrophosphate dehydrate deposition disease. MSUS, musculoskeletal ultrasonography; PMR, polymyalgia rheumatica; RA, rheumatoid arthritis.

Common symptoms and findings of inflammatory arthritis include joint swelling, erythema, and reduced joint range of motion. However, it can be difficult to determine which musculoskeletal part of the joint is the center of inflammation based only on physical examination. Clinicians can comprehensively evaluate musculoskeletal structures using ultrasonography with a high resolution and thereby identify the anatomical location that is mainly affected. This is useful for formulating differential diagnoses of inflammatory arthritis.^[14, 23,24,25]^ We summarize the MSUS pathological findings in Figure 2 and their corresponding differential diagnoses in Table 3.

**Table 3:** MSUS findings and corresponding differential diagnosis of inflammatory arthritis

**Location**	**MSUS findings**	**Differential diagnosis**
**Joint recess**	Synovitis	RA>SpA
		CTDs (SLE, SSc, myositis, AAV)
**Bursa**	Bursitis	PMR
		RA (with synovial hypertrophy)
		Crystal or septic arthritis etc.
**Tendon**	Tenosynovitis	RA, PMR, SpA, CTDs
	Enthesitis	SpA (PsA, AS, IBD, etc.), CTDs
	Tendinitis	SpA, CTDs (SLE, Becet, SSc, etc)
**Bone**	Erosion	RA, Gout, etc.
	Osteophyte	OA, PsA, SpA, etc.
**Cartilage**	Cartilage loss	OA, etc.
	Crystal deposit	Gout, pseudo gout, HADD

MSUS, musculoskeletal ultrasonography; RA, rheumatoid arthritis; SpA, spondyloarthropathitis; CTD, connective tissue disease; SLE, systemic lupus erythematosus; SSc, systemic sclerosis; AAV, anti-neutrophil cytoplasmic antibody-associated vasculitis; PMR, polymyalgia rheumatica; PsA, psoriatic arthritis; AS, ankylosing spondylitis; IBD, inflammatory bowel disease; OA, osteoarthritis HADD, hydroxyapatite deposition disease.

### Enthesitis

A characteristic feature of SpA is inflammation at the tendon or ligament attachment sites. The entheses and an anatomically pathological hallmark of inflammatory rheumatic diseases have inflammatory processes involving the synovioentheseal sites.^[26]^ The inflammatory microenvironment of the synovio-entheseal complex, named enthesitis, is characterized by an initial inflammatory or erosive phase, which has important implications for understanding pathological changes in MSUS.^[27, 28]^ Enthesitis may be hyposymptomatic and difficult to identify on physical examination alone.^[23]^ However, it can be detected sensitively using MSUS. Balint *et al*.^[29]^ showed that MSUS was used to detect enthesitis in 56% of sites compared with clinical examination, which only detected enthesitis in 22% of sites.^[29]^ Ultrasound has also been useful in depicting nail diseases in PsA patients. Psoriasis is associated with distal interphalangeal enthesopathy.^[30]^

**Figure 3:**
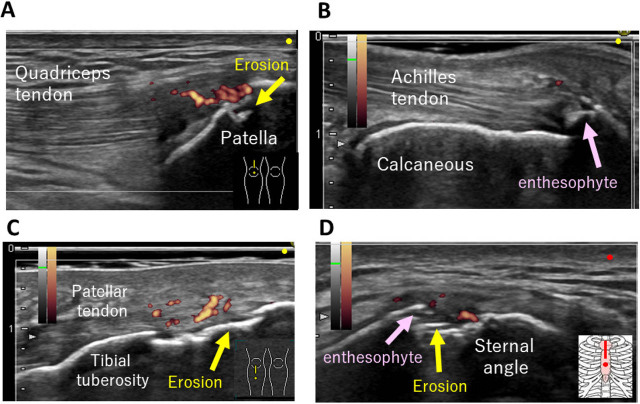
Various enthesitis findings detected by MSUS in SpA patients. (A) Longitudinal view of active enthesitis with power Doppler signals and erosion of the quadriceps tendon in a patient with PsA. (B) Chronic enthesitis with enthesophyte of the Achilles tendon. (C) Patellar tendon active enthesitis from a patient with reactive arthritis. (D) Active enthesitis on the manubriosternal joint in a patient with inflammatory bowel disease–associated arthritis. MSUS, musculoskeletal ultrasonography; PsA, psoriatic arthritis; SpA, spondyloarthritis.

Sonographic enthesitis is defined as the loss of fibrillar echotexture, tendon thickening, and hypervascularity within 2 mm of the bony cortex, as proposed by the Outcome Measures in Rheumatology ultrasound subgroup.^[31]^ Chronic enthesitis or degenerative changes may manifest as tendon thickening, bulky enthesophytes, intratendinous calcification, and bone erosions (Figure 3).^[32, 33]^ In addition, ultrasound enthesitis scoring systems, including the Glasgow Ultrasound Enthesitis Scoring System and the Madrid Sonographic Enthesitis Index (MASEI), have been proposed.^[34, 35]^ These scoring systems were validated as tools for the diagnostic classification of SpA.^[36, 37]^ In a recent cross-sectional study involving 113 patients with early SpA and 57 matched controls, a MASEI cutoff score of ≥20 had a specificity of 89.5% in differentiating between patients with SpA and healthy controls.^[37]^ This is because degenerative or mechanical abnormalities in weight-bearing joints may be incorrectly identified as joint enthesopathy in control subjects, and these scores are associated with age and body mass index.^[34, 38, 39]^ Considering their effort- and time-intensive nature, these scoring systems are mainly used for clinical research and are not used in routine daily practice.

Functional enthesis, proposed by Benjamin and McGonagle^[28]^ in 2007, is a similar region of tendon–bone contact distant from the major attachment site. It is distributed around the tendons and ligaments of the digits, peroneal tendons, and tibialis posterior of the ankle.^[40]^ Similar to the enthesis, tendon–bone contact at a functional enthesis corresponds to increased levels of shear and/or compression, leading to the differentiation of fibrocartilage. Functional entheses are the sites of pathology in SpA; thus, tenosynovitis, dactylitis, and peri-tendon inflammation are well-recognized features. Recent reports showed that extensor tendon tendonitis, extensor slip enthesitis, and periarticular edema are useful in differentiating PsA from RA.^[24, 25]^ However, a recent report showed that periextensor tendon inflammation may be found in patients with SLE,^[41]^ and we also noted it in patients with other phenotypes of inflammatory arthritis, including immune-related adverse events, after using immune checkpoint inhibitors.

### Bursitis

Determining the differential diagnoses of polymyalgia rheumatica (PMR) and RA in older patients with shoulder and hip pain is still challenging because there are no specific serological or imaging biomarkers suggestive of these conditions.^[42, 43]^ The provisional classification criteria for PMR by the EULAR/ACR have proposed ultrasound features suggestive of a differential diagnosis of PMR. These include bilateral subacromial and subdeltoid bursitis, long biceps tendon tenosynovitis, trochanteric bursitis, and glenohumeral and hip effusion.^[44]^ However, adding ultrasound items to the scoring system did not increase the sensitivity and slightly increased the specificity for discriminating PMR from other mimicking conditions, such as elderly onset RA (EORA), from 78% to 81%. This may have been caused by the limited sensitivity of ultrasound in detecting abnormalities in the hip compared with MRI in PMR.^[44]^

In another study, bilateral shoulder bursitis on ultrasound had the highest specificity for any individual in determining the diagnosis of PMR.^[45, 46]^ The quality of bursitis diagnosis can be useful when considering differential diagnoses. Suzuki *et al*.^[47]^ recently showed that moderate-to-severe proliferative synovitis of the shoulder bursae, especially in the subacromial bursa, is a key feature for discriminating EORA from PMR. Focusing on MSUS findings rather than bursitis is also important, and the absence of active synovitis in the hand or wrist on ultrasound is suggestive of PMR rather than EORA.^[26]^ A recent study also showed that tenosynovitis, tendinitis, and ligament inflammation in the shoulder and knee detected by MSUS assessment may improve the accuracy of the 2012 EULAR/ACR provisional classification criteria for PMR.^[48]^

### Cartilage and Crystal Deposits

MSUS can diagnose and distinguish crystal-induced arthropathies, although the traditional “gold standard” for diagnosis is based on direct microscopic observation of each crystal. Characteristic ultrasonographic findings in crystal-induced arthropathies can be divided into non-specific inflammation (including synovitis, tenosynovitis, and extra-articular soft tissue inflammation) and crystal deposition.^[49]^ MSUS can uniquely distinguish between crystal deposits of monosodium urate crystal deposition in gout on the cartilage surface, called the double contour (DC) sign, and intra-cartilaginous chondrocalcinosis of calcium pyrophosphate crystal deposit disease (CPPD).^[50, 51]^ However, the DC sign is not present in all patients with gout, and studies have shown extremely varying sensitivities ranging from 0.22 to 0.92.^[49]^ Furthermore, a previous study showed that the DC sign alone is suitable for predicting crystal-induced arthropathies, but it cannot reliably distinguish gout from CPPD in routine clinical practice.^[52]^ This seems to be because the perpendicular ultrasound beams are reflected as a result of impedance differences at the boundary of the cartilage surface and may be mistaken for the DC sign. Additionally, even in CPPD, intra-chondral calcification may appear on the cartilage surface if it has low thickness or if calcification is severe, and such circumstances can also pose a challenge in detecting the DC sign. Importantly, in routine clinical practice, MSUS provides important information to distinguish gout from CPPD; however, its usefulness is enhanced when combined with clinical conditions, such as age and serum uric acid levels.

## Conclusion

With the increasing use of molecular target–specific therapies, including biologic agents and JAK inhibitors, it has become more important to establish an early and accurate diagnosis of inflammatory rheumatic diseases. Consequently, novel imaging modalities, including MSUS as a predominant example, have been developed to fulfill an important role in the diagnosis of rheumatic diseases. They can be used to detect subclinical inflammation and to identify the accurate distribution of joint involvement and inflammation sites in each joint. MSUS provides further opportunities and motivation to consider other differential diagnoses, especially in patients with sparse clinical and laboratory signs of inflammation.
